# Supplementary material for: Demographic, Knowledge and Impact Analysis of 57,627 Antibiotic Guardians Who Have Pledged to Contribute to Tackling Antimicrobial Resistance
Source: Antibiotics (Basel). 2019 Mar 9;8(1):21. doi: 10.3390/antibiotics8010021 (PMC6466561; doi:10.3390/antibiotics8010021)
Supplement: Supplementary file 1 [file antibiotics-08-00021-s001.zip › S1 Table.pdf]

S1 Table. Antibiotic Guardian rate by CCG per 100,000 population with 95% confidence intervals

CCG Code	CCG	Rates per 100,000	LowerCI	UpperCI
06H	NHS Castle Point and Rochford CCG	9	5	15
00J	NHS Nottingham North and East CCG	16	10	24
06V	NHS North Norfolk CCG	16	11	23
04R	NHS Thurrock CCG	23	16	31
02A	NHS West Norfolk CCG	23	17	32
05J	NHS South Lincolnshire CCG	23	16	32
09F	NHS Great Yarmouth and Waveney CCG	25	19	32
12F	NHS Fylde and Wyre CCG	26	19	34
01F	NHS Havering CCG	27	21	34
06L	NHS Lincolnshire East CCG	28	22	36
05R	NHS Swale CCG	29	20	41
04F	NHS North Lincolnshire CCG	30	22	39
99F	NHS Corby CCG	30	19	46
04N	NHS South Tyneside CCG	31	23	41
14L	NHS North East Essex CCG	31	25	38
10Q	NHS South East Staffordshire and Seisdon Peninsula CCG	31	25	40
05D	NHS Fareham and Gosport CCG	32	24	41
09W	NHS North East Lincolnshire CCG	32	24	42
02P	NHS Barnsley CCG	33	26	41
02G	NHS South Norfolk CCG	33	26	41
06D	NHS Morecambe Bay CCG	34	28	41
99E	NHS Basildon and Brentwood CCG	34	27	42
10R	NHS South Eastern Hampshire CCG	35	27	44
02Y	NHS Erewash CCG	35	24	49
15A	NHS Bexley CCG	36	29	44
09A	NHS Coventry and Rugby CCG	36	31	42
08N	NHS South Kent Coast CCG	37	29	46
04E	NHS North East Hampshire and Farnham CCG	37	30	47
10X	NHS Telford and Wrekin CCG	38	29	48
08T	NHS Warwickshire North CCG	39	31	49
08R	NHS North Hampshire CCG	39	31	48
05W	NHS Walsall CCG	39	32	47
05A	NHS Doncaster CCG	40	33	48
09N	NHS Hastings and Rother CCG	40	31	50
03W	NHS Enfield CCG	40	33	47
02Q	NHS Bassetlaw CCG	40	30	54
00C	NHS Durham Dales, Easington and Sedgefield CCG	41	34	49
05N	NHS Southend CCG	42	33	52
06M	NHS Haringey CCG	42	35	51
01T	NHS Surrey Downs CCG	44	36	52
15F	NHS Mid Essex CCG	45	38	52
06F	NHS Bedfordshire CCG	45	39	52
03M	NHS South Worcestershire CCG	45	38	53
02N	NHS Airedale, Wharfedale and Craven CCG	45	35	57
08X	NHS Wyre Forest CCG	46	33	61

05L	NHS South West Lincolnshire CCG	46	35	59
00L	NHS Scarborough and Ryedale CCG	46	35	61
03Q	NHS West Suffolk CCG	46	38	56
00X	NHS Crawley CCG	47	35	61
12D	NHS West Essex CCG	47	39	55
02T	NHS Canterbury and Coastal CCG	47	38	57
15D	NHS Eastbourne, Hailsham and Seaford CCG	47	38	58
05X	NHS West Kent CCG	47	41	54
10K	NHS Hambleton, Richmondshire and Whitby CCG	48	37	60
07T	NHS Croydon CCG	48	41	56
07G	NHS West Leicestershire CCG	48	42	55
09H	NHS Dorset CCG	49	44	54
02H	NHS East Berkshire CCG	49	43	56
03X	NHS Halton CCG	49	38	63
08A	NHS Hartlepool and Stockton-on-Tees CCG	50	42	59
04D	NHS Newham CCG	50	43	58
00P	NHS Waltham Forest CCG	51	43	60
01G	NHS South Warwickshire CCG	51	43	60
08E	NHS Horsham and Mid Sussex CCG	51	43	61
10E	NHS West Lancashire CCG	52	39	67
03T	NHS Newark and Sherwood CCG	52	40	67
10J	NHS Portsmouth CCG	52	43	63
08D	NHS High Weald Lewes Havens CCG	52	42	64
08K	NHS Merton CCG	53	43	64
01J	NHS Medway CCG	53	45	62
00T	NHS Bradford Districts CCG	53	46	61
05F	NHS Isle of Wight CCG	53	42	67
03Y	NHS Heywood, Middleton and Rochdale CCG	54	45	65
07P	NHS Bromley CCG	54	46	63
09P	NHS Hull CCG	54	46	64
06T	NHS Nottingham West CCG	54	41	70
08L	NHS Nene CCG	55	49	60
10L	NHS Lincolnshire West CCG	55	46	65
09C	NHS Ashford CCG	55	43	69
07K	NHS Buckinghamshire CCG	55	49	62
01R	NHS Southport and Formby CCG	55	43	71
09E	NHS City and Hackney CCG	55	47	65
05T	NHS Tameside and Glossop CCG	56	47	65
06Y	NHS Sunderland CCG	56	47	65
03E	NHS Hillingdon CCG	56	47	65
06P	NHS North Durham CCG	56	47	66
00K	NHS Hounslow CCG	56	48	66
00Q	NHS Bolton CCG	56	48	66
09L	NHS Gloucestershire CCG	57	51	63
13T	NHS North West Surrey CCG	57	49	65
03J	NHS Redbridge CCG	57	49	66
09J	NHS Ealing CCG	58	50	67
99C	NHS North Tyneside CCG	58	48	70
00D	NHS East Riding of Yorkshire CCG	58	50	67
08F	NHS Ipswich and East Suffolk CCG	59	51	66

03V	NHS Dartford, Gravesham and Swanley CCG	59	50	69
02D	NHS Wigan Borough CCG	59	51	68
08C	NHS Herts Valleys CCG	59	53	66
11X	NHS Southern Derbyshire CCG	61	54	68
05G	NHS Rotherham CCG	61	52	71
06W	NHS Sheffield CCG	61	55	68
07L	NHS Barking and Dagenham CCG	62	52	73
10A	NHS Stockport CCG	62	53	72
08G	NHS Knowsley CCG	64	52	78
03F	NHS Lewisham CCG	65	56	74
00Y	NHS South Devon and Torbay CCG	65	56	75
01Y	NHS West Hampshire CCG	65	59	72
09X	NHS Lambeth CCG	66	57	75
04Q	NHS Swindon CCG	66	56	77
07N	NHS Blackburn with Darwen CCG	66	53	80
00L	NHS Northumberland CCG	67	58	76
07Q	NHS Cambridgeshire and Peterborough CCG	67	62	73
15E	NHS Blackpool CCG	68	55	83
04L	NHS Somerset CCG	68	62	75
05H	NHS Coastal West Sussex CCG	68	61	76
99A	NHS North Derbyshire CCG	69	59	79
06K	NHS East Surrey CCG	69	57	82
07X	NHS Greenwich CCG	69	60	79
02E	NHS Newcastle Gateshead CCG	69	62	77
08P	NHS South Sefton CCG	70	58	84
09Y	NHS Salford CCG	70	60	82
03L	NHS South Tees CCG	72	62	83
07H	NHS North Cumbria CCG	72	63	82
01X	NHS Vale of York CCG	72	64	82
05C	NHS East Leicestershire and Rutland CCG	75	66	85
00R	NHS Bradford City CCG	75	58	96
05V	NHS Vale Royal CCG	76	61	95
11M	NHS Hardwick CCG	76	61	94
03D	NHS Herefordshire CCG	77	65	90
99C	NHS Rushcliffe CCG	78	62	95
11J	NHS East Lancashire CCG	80	71	90
99J	NHS Norwich CCG	81	69	94
04K	NHS Shropshire CCG	81	72	92
02W	NHS Brent CCG	84	74	94
08M	NHS Northern, Eastern and Western Devon CCG	84	78	90
99M	NHS Oldham CCG	84	73	96
07J	NHS Bristol, North Somerset and South Gloucestershire CCG	84	79	90
05L	NHS Sandwell and West Birmingham CCG	85	77	93
00M	NHS Surrey Heath CCG	86	68	106
01X	NHS St Helens CCG	87	74	102
09D	NHS Bury CCG	89	76	103
10V	NHS Stafford and Surrounds CCG	90	76	106
10C	NHS Warrington CCG	91	79	105
07M	NHS Barnet CCG	93	83	103

04J	NHS Nottingham City CCG	93	83	104
00V	NHS Cannock Chase CCG	93	78	111
99H	NHS Wandsworth CCG	94	83	105
01V	NHS Tower Hamlets CCG	94	84	106
08J	NHS Mansfield and Ashfield CCG	97	84	112
02R	NHS Brighton and Hove CCG	98	86	110
06N	NHS Islington CCG	98	86	111
02X	NHS East and North Hertfordshire CCG	100	92	108
04M	NHS South Cheshire CCG	100	86	116
99N	NHS Leeds CCG	101	94	108
02F	NHS Guildford and Waverley CCG	104	91	119
01W	NHS Wakefield CCG	104	94	116
15C	NHS Calderdale CCG	105	92	120
99G	NHS Thanet CCG	107	90	125
08Q	NHS Trafford CCG	107	95	122
03A	NHS Harrogate and Rural District CCG	108	93	125
03K	NHS Redditch and Bromsgrove CCG	108	94	124
03R	NHS Wiltshire CCG	112	103	122
07R	NHS Central London (Westminster) CCG	112	97	129
03H	NHS Oxfordshire CCG	112	105	121
99K	NHS Kingston CCG	113	98	130
15A	NHS Berkshire West CCG	116	106	125
09G	NHS Darlington CCG	118	98	140
01A	NHS Eastern Cheshire CCG	120	105	136
01E	NHS Harrow CCG	122	109	137
01D	NHS Kernow CCG	125	116	135
04C	NHS Milton Keynes CCG	126	113	140
00N	NHS Sutton CCG	126	111	143
05Y	NHS Wirral CCG	128	116	141
06V	NHS Richmond CCG	133	117	150
06A	NHS Greater Preston CCG	134	118	151
06Q	NHS North Kirklees CCG	137	121	155
07W	NHS East Staffordshire CCG	140	120	162
07Y	NHS Leicester City CCG	148	136	161
04G	NHS North Staffordshire CCG	155	139	172
10D	NHS West Cheshire CCG	160	144	177
04V	NHS Birmingham and Solihull CCG	160	153	167
08H	NHS Liverpool CCG	163	152	174
07V	NHS Dudley CCG	165	151	179
08V	NHS West London CCG	166	149	184
04Y	NHS Chorley and South Ribble CCG	171	152	191
08W	NHS Wolverhampton CCG	172	156	189
99D	NHS Stoke on Trent CCG	199	182	217
14Y	NHS Camden CCG	201	183	219
08C	NHS Hammersmith and Fulham CCG	204	184	226
11N	NHS Luton CCG	242	222	264
01C	NHS Greater Huddersfield CCG	245	226	265
11A	NHS Manchester CCG	253	240	267
03N	NHS Southampton CCG	270	250	291
99Q	NHS Southwark CCG	324	305	345

11E	NHS Bath and North East Somerset CCG	458	428	490
