# Supplementary material for: Demographic, Knowledge and Impact Analysis of 57,627 Antibiotic Guardians Who Have Pledged to Contribute to Tackling Antimicrobial Resistance
Source: Antibiotics (Basel). 2019 Mar 9;8(1):21. doi: 10.3390/antibiotics8010021 (PMC6466561; doi:10.3390/antibiotics8010021)
Supplement: Supplementary file 1 [file antibiotics-08-00021-s001.zip › S2 Table.pdf]

S2 Table. Antibiotic Guardian rate by local authority per 100,000 population with 95% confidence intervals

**Local Authority**	**Rate Per 100,000**	**LowerCI**	**UpperCI**
Adur	52	36	73
Allerdale	46	34	62
Amber Valley	27	19	38
Arun	57	46	70
Ashfield	143	123	166
Ashford	55	43	69
Aylesbury Vale	48	39	59
Babergh	22	13	34
Barking and Dagenham	33	26	42
Barnet	120	110	132
Barnsley	39	32	48
Barrow-in-Furness	33	21	50
Basildon	27	20	35
Basingstoke and Deane	38	30	49
Bassetlaw	69	55	86
Bath and North East Somerset	458	428	490
Bedford	50	40	62
Bexley	36	29	44
Birmingham	185	177	193
Blaby	126	105	150
Blackburn with Darwen	66	53	80
Blackpool	68	55	83
Bolsover	143	118	172
Bolton	48	41	57
Boston	29	18	45
Bournemouth	49	39	60
Bracknell Forest	38	28	51
Bradford	51	45	57
Braintree	28	20	37
Breckland	17	11	26
Brent	41	34	48
Brentwood	46	32	64
Brighton and Hove	98	87	110
Bristol, City of	111	101	121
Broadland	42	32	55
Bromley	52	45	61
Bromsgrove	70	54	88
Broxbourne	65	50	83
Broxtowe	16	9	25
Burnley	40	28	56
Bury	100	86	116
Calderdale	105	92	120
Cambridge	82	67	99
Camden	242	224	262

Cannock Chase	63	48	80
Canterbury	53	42	65
Carlisle	45	33	60
Castle Point	9	4	18
Central Bedfordshire	44	37	52
Charnwood	117	102	134
Chelmsford	69	57	82
Cheltenham	77	62	94
Cherwell	57	45	70
Cheshire East	112	102	123
Cheshire West and Chester	132	120	145
Chesterfield	34	24	48
Chichester	86	70	104
Chiltern	57	43	74
Chorley	46	34	60
Christchurch	18	8	34
City of London	314	201	467
Colchester	51	41	62
Copeland	23	13	38
Corby	30	19	46
Cornwall	122	113	132
Cotswold	48	35	65
County Durham	43	38	49
Coventry	41	34	48
Craven	67	48	92
Crawley	47	35	61
Croydon	45	39	53
Dacorum	28	20	38
Darlington	138	117	162
Dartford	100	82	121
Daventry	13	7	24
Derby	64	54	74
Derbyshire Dales	111	88	139
Doncaster	35	29	43
Dover	28	19	39
Dudley	147	134	161
Ealing	60	52	69
East Cambridgeshire	86	67	107
East Devon	44	34	57
East Dorset	29	19	43
East Hampshire	37	27	49
East Hertfordshire	60	48	74
East Lindsey	46	35	58
East Northamptonshire	6	2	14
East Riding of Yorkshire	55	47	64
East Staffordshire	171	148	196
Eastbourne	54	41	70
Eastleigh	35	26	47
Eden	53	35	77
Elmbridge	33	24	44

Enfield	38	32	46
Epping Forest	44	34	57
Epsom and Ewell	76	58	97
Erewash	17	11	27
Exeter	148	128	171
Fareham	52	40	67
Fenland	32	22	45
Forest Heath	11	4	22
Forest of Dean	52	38	70
Fylde	24	15	38
Gateshead	31	23	39
Gedling	33	24	46
Gloucester	67	54	83
Gosport	32	21	46
Gravesham	50	37	65
Great Yarmouth	27	18	40
Greenwich	57	49	67
Guildford	120	103	140
Hackney	64	55	74
Halton	49	38	63
Hambleton	56	42	74
Hammersmith and Fulham	186	167	207
Harborough	49	36	66
Haringey	42	35	51
Harlow	50	36	67
Harrogate	104	89	121
Harrow	120	107	134
Hart	37	26	51
Hartlepool	35	24	50
Hastings	50	36	66
Havant	38	28	51
Havering	51	43	61
Herefordshire, County of	85	72	99
Hertsmere	37	26	50
High Peak	52	38	69
Hillingdon	51	44	60
Hinckley and Bosworth	24	16	35
Horsham	49	38	62
Hounslow	59	51	69
Huntingdonshire	74	62	88
Hyndburn	179	151	211
Ipswich	91	76	108
Isle of Wight	53	42	67
Isles of Scilly	133	27	388
Islington	72	61	84
Kensington and Chelsea	162	142	183
Kettering	97	78	118
King's Lynn and West Norfolk	24	17	33
Kingston upon Hull, City of	56	48	66
Kingston upon Thames	104	90	121

Kirklees	198	185	211
Knowsley	69	57	84
Lambeth	79	70	90
Lancaster	64	51	78
Leeds	104	97	111
Leicester	125	114	138
Lewes	63	48	80
Lewisham	70	61	80
Lichfield	56	43	72
Lincoln	101	82	122
Liverpool	135	125	145
Luton	242	222	264
Maidstone	61	50	74
Maldon	27	15	43
Malvern Hills	35	23	51
Manchester	261	247	275
Mansfield	23	15	34
Medway	48	40	57
Melton	108	81	141
Mendip	95	78	115
Merton	53	43	64
Mid Devon	41	28	57
Mid Suffolk	34	24	48
Mid Sussex	45	35	57
Middlesbrough	108	92	127
Milton Keynes	124	111	138
Mole Valley	48	35	65
New Forest	67	56	81
Newark and Sherwood	31	22	42
Newcastle upon Tyne	96	85	107
Newcastle-under-Lyme	179	157	204
Newham	25	20	31
North Devon	175	149	204
North Dorset	58	41	78
North East Derbyshire	68	53	87
North East Lincolnshire	31	23	41
North Hertfordshire	64	51	79
North Kesteven	17	11	27
North Lincolnshire	37	28	47
North Norfolk	17	10	27
North Somerset	93	80	106
North Tyneside	58	48	70
North Warwickshire	16	7	29
North West Leicestershire	77	61	96
Northampton	81	69	93
Northumberland	70	61	80
Norwich	92	77	109
Nottingham	104	93	116
Nuneaton and Bedworth	44	34	57
Oadby and Wigston	46	30	67

Oldham	87	76	100
Oxford	295	269	323
Pendle	73	56	93
Peterborough	40	32	50
Plymouth	77	67	89
Poole	67	54	81
Portsmouth	52	43	63
Preston	205	182	230
Purbeck	28	15	48
Reading	245	222	271
Redbridge	79	69	90
Redcar and Cleveland	35	26	47
Redditch	29	19	43
Reigate and Banstead	94	79	111
Ribble Valley	50	34	72
Richmond upon Thames	126	110	142
Richmondshire	41	26	62
Rochdale	54	45	65
Rochford	9	4	18
Rossendale	38	25	56
Rother	27	18	40
Rotherham	48	40	57
Rugby	22	14	32
Runnymede	87	69	109
Rushcliffe	21	13	31
Rushmoor	58	44	76
Rutland	58	37	87
Ryedale	28	15	46
Salford	77	67	89
Sandwell	64	56	74
Scarborough	44	33	59
Sedgemoor	32	23	44
Sefton	101	89	113
Selby	16	9	27
Sevenoaks	42	31	55
Sheffield	65	59	72
Shepway	42	31	56
Shropshire	81	71	91
Slough	30	22	40
Solihull	57	47	68
South Bucks	63	46	85
South Cambridgeshire	123	106	142
South Derbyshire	52	39	68
South Gloucestershire	42	34	50
South Hams	33	22	47
South Holland	16	9	27
South Kesteven	41	31	53
South Lakeland	66	51	84
South Norfolk	51	40	64
South Northamptonshire	43	30	59

South Oxfordshire	42	32	54
South Ribble	224	197	253
South Somerset	67	55	81
South Staffordshire	72	57	90
South Tyneside	31	23	41
Southampton	270	250	291
Southend-on-Sea	42	33	52
Southwark	306	286	325
Spelthorne	26	17	38
St Albans	58	47	72
St Edmundsbury	50	38	65
St. Helens	103	88	119
Stafford	98	82	116
Staffordshire Moorlands	98	80	120
Stevenage	206	177	239
Stockport	74	65	85
Stockton-on-Tees	56	46	68
Stoke-on-Trent	210	192	228
Stratford-on-Avon	23	16	33
Stroud	50	38	64
Suffolk Coastal	36	27	48
Sunderland	56	47	65
Surrey Heath	83	65	105
Sutton	126	111	143
Swale	31	22	41
Swindon	54	45	65
Tameside	56	46	66
Tamworth	42	29	59
Tandridge	19	11	31
Taunton Deane	91	75	110
Teignbridge	37	27	48
Telford and Wrekin	40	31	50
Tendring	19	13	28
Test Valley	87	71	105
Tewkesbury	34	23	49
Thanet	107	90	125
Three Rivers	51	37	67
Thurrock	23	16	31
Tonbridge and Malling	53	41	67
Torbay	86	71	103
Torridge	60	43	82
Tower Hamlets	107	96	120
Trafford	63	53	74
Tunbridge Wells	28	19	39
Uttlesford	41	29	57
Vale of White Horse	69	55	84
Wakefield	97	86	108
Walsall	52	44	61
Waltham Forest	45	38	54
Wandsworth	73	64	83

Warrington	97	84	112
Warwick	76	62	91
Watford	154	130	181
Waveney	25	17	36
Waverley	54	42	68
Wealden	47	37	59
Wellingborough	35	24	51
Welwyn Hatfield	108	90	128
West Berkshire	38	29	49
West Devon	87	64	115
West Dorset	65	50	82
West Lancashire	52	39	67
West Lindsey	3	1	9
West Oxfordshire	57	44	73
West Somerset	26	12	49
Westminster	133	119	148
Weymouth and Portland	18	9	32
Wigan	59	51	68
Wiltshire	113	104	123
Winchester	70	56	87
Windsor and Maidenhead	76	63	91
Wirral	128	116	141
Woking	62	48	80
Wokingham	58	47	70
Wolverhampton	151	137	167
Worcester	60	46	77
Worthing	73	58	91
Wychavon	64	51	79
Wycombe	38	30	49
Wyre	27	18	39
Wyre Forest	37	26	51
York	93	80	107
